# Supplementary material for: Risk factors for surgical site infections using a data-driven approach
Source: PLoS One. 2020 Oct 28;15(10):e0240995. doi: 10.1371/journal.pone.0240995 (PMC7592760; doi:10.1371/journal.pone.0240995)
Supplement: S1 Table — (DOCX) [file pone.0240995.s001.docx]

**S1 Table. Risk factors identified from multivariate analysis during literature search.**

| Risk Factor | Significance^1^ | Digestive System^2^ | Orthopaedic^2^ | Thoracic^2^ |
| --- | --- | --- | --- | --- |
| Age* | D_U_,T_M_ | [14,15,34,38] | [21] | [18] |
| Alcohol use* | O_U_,T_U_ |  | [42] |  |
| Allergy history |  |  | [42] |  |
| Anaesthesia |  |  | [21] |  |
| Antibiotic use* | ­­D_M_,O_M_,T_M_ |  |  | [17] |
| Antimicrobial prophylaxis |  |  | [42] |  |
| ASA Class* | O_U_ | [13,16,32,34,45] | [21,42,44] | [21] |
| Blood loss |  |  | [44] |  |
| BMI* | None | [35] | [42–44] | [33] |
| Cardiac failure |  |  |  | [33] |
| Catecholamine duration |  |  |  | [17] |
| COPD |  |  | [36] |  |
| Colostomy |  | [14] |  |  |
| Critical preoperative status |  |  |  | [17] |
| CRP* | D_M_ |  | [21] |  |
| Diabetes* | None | [14,38,41] | [21,36,42,44] | [19] |
| Dialysis |  | [40] |  | [17] |
| Drainage use |  | [32] | [21,42] |  |
| Duration of aortic clamping |  |  |  | [17] |
| Duration of mechanical ventilation |  |  |  | [19] |
| Duration of surgery* | D_M_ | [11,14,32,34,35,40,45] | [21,36,42,44] |  |
| EBL |  | [37] |  |  |
| Emergency* | Excluded from analysis | [14,32,34,38] |  | [17,33] |
| EuroSCORE |  |  |  | [17] |
| External fixator use |  |  | [42] |  |
| Gender* | D_U_ | [14,15,34] | [21,42] |  |
| Glucose* | None | [38] |  |  |
| Haemoglobin* | None | [15,35,45] |  |  |
| Heart Disease* | None |  | [42] | [18] |
| High-energy mechanism |  |  | [42] |  |
| Hypertension* | None |  | [42] |  |
| ICU admission history |  |  |  | [17–19] |
| Immunosuppression |  |  | [21] | [17,33] |
| Incision cleanness |  |  | [36,42] |  |
| Laparoscopy |  | [14,34] |  |  |
| Leukocyte* | None | [46] |  |  |
| Liver disease* | None | [45] |  |  |
| NNIS |  | [38] |  |  |
| Open fracture |  |  | [42] |  |
| Preoperative length of stay* | D_U_ | [32,41] | [21,43] | [17–19] |
| Previous hospitalisation |  |  |  | [17] |
| Previous SSI |  |  | [44] |  |
| Previous surgery |  |  | [42,44] |  |
| Race |  |  | [21] |  |
| Renal impairment |  |  |  | [33] |
| Serum sodium |  | [40] |  |  |
| Serum total protein* | None | [11,40] |  |  |
| Smoking* | None | [40] | [42–44] |  |
| Steroids |  | [14] |  |  |
| Subluxation/dislocation |  |  | [42] |  |
| Surgeon experience |  | [35] | [42] |  |
| Temperature* | D_M_,O_M_,T_M_ | [46] |  |  |
| Thrombocyte |  | [15] |  |  |
| Total bilirubin |  | [40] |  |  |
| Transfusion |  | [14,32,37] |  |  |
| Type of surgery* | Used to segment data | [32,37] | [21,44] |  |
| Unimalleolar |  |  | [42] |  |
| Weber C type |  |  | [42] |  |
| Wound class |  | [14,32,34] |  |  |

D, Digestive system surgical procedures; O, Orthopaedic system surgical procedures; T, Thoracic system surgical procedures; _U_, Significant in univariate analysis; _M_, Significant in multivariate analysis; ASA, American Society of Anaesthesiologists; CRP, C-reactive protein; SSI, Surgical Site Infection; BMI, Body Mass Index; COPD, Chronic obstructive pulmonary disease; NNIS, Surgical Infection Risk Index.

^1^During which part of the analysis the risk factor was found statistically significant.

^2^References to the literature which had the risk factor as a multivariate result for each group of surgeries.

^*^Present in data.
