# Supplementary material for: Risk factors for surgical site infections using a data-driven approach
Source: PLoS One. 2020 Oct 28;15(10):e0240995. doi: 10.1371/journal.pone.0240995 (PMC7592760; doi:10.1371/journal.pone.0240995)
Supplement: S1 Formulae — (DOCX) [file pone.0240995.s002.docx]

**S2 Formulae. The multivariate logistic regression equations based on the data-driven cut-offs.**

The multivariate logistic regression equations, based on the data-driven cut-offs, are provided in Equation 1 to Equation 3.

$$P\left( SSI=1|Digestive System Surgical Procedure \right)=\left( 1+exp-(-4.983+1.067\times\mathrm{Ind}\left[ 38<TEM\leq39 \right]+1.732\times\mathrm{Ind}\left[ TEM>39 \right]+1.201\times ANT+0.002\times DUR+0.639\times\mathrm{Ind}\left[ CRP>8.1 \right]) \right)^{-1} (1)$$

$P\left( SSI=1|Orthopeadic Surgical Procedure \right)=\left( 1+exp-(-19.918+1.552\times ANT+1.224\times\mathrm{Ind}\left[ TEM>39 \right]) \right)^{-1} (2)$

$$P\left( SSI=1|Thoracic Surgical Procedure \right)=\left( 1+exp-(-3.101+0.824\times\mathrm{Ind}\left[ TEM>38 \right]-1.847\times\mathrm{Ind}\left[ AGE>17 \right]+1.597\times ANT) \right)^{-1} (3)$$

Where $TEM$ = highest temperature of patient in the past 7 days before surgery, $ANT$ = antibiotic use of patient at the time of surgery (yes/no), $DUR$ = duration of the surgical procedure (minutes), $CRP$ = highest CRP of patient in the 7 days before surger, $AGE$ = age of patient on the day of surgery (years) and $\mathrm{Ind}\left[ logic statement \right]=1$ if the logic statement is true and $0$ if it is false.
