# Supplementary material for: Risk factors for surgical site infections using a data-driven approach
Source: PLoS One. 2020 Oct 28;15(10):e0240995. doi: 10.1371/journal.pone.0240995 (PMC7592760; doi:10.1371/journal.pone.0240995)
Supplement: Attachment — Submitted filename: Response to Reviewers.docx [file pone.0240995.s003.docx]

**Response to reviewers – PONE-D-20-14495**

Risk factors for surgical site infections using a data-driven approach

Johan Magnus van Niekerk, Margreet C. Vos, Alfred Stein, Annemarie Braakman-Jansen, Anne F. Voor in ’t holt, Lisette van Gemert-Pijnen

**Editor**, Dr. Francesco Di Gennaro,

*Thank you for giving us the opportunity to revise our manuscript. Please find our response to the reviewers’ comments below.*

**Journal requirements:**

1. Please ensure that your manuscript meets PLOS ONE's style requirements, including those for file naming.

*The PLOS ONE journal style documents were carefully perused; adjustments were made throughout the manuscript accordingly.*

2. We note that you have provided information about your ethics approval and data anonymization in the Ethics Statement. We ask that you additionally provide this information in your Methods section.

*L139-L140: The ethics approval and data anonymization statements were added to the Methods section.*

3. We note that you have indicated that data from this study are available upon request. PLOS only allows data to be available upon request if there are legal or ethical restrictions on sharing data publicly.

*Even in its anonymised format, the data used in this study may still lead to the discovery of the patient’s identity. These data contain the exact date and time which the surgery took place, the type of surgery, the gender and age of the patient and well as the patient’s comorbidities. The medical ethical technical committee of Erasmus MC did not grant permission to publish these data due to ethical considerations and the sensitivity of the data. Data are however available from the Erasmus MC upon reasonable request.*

*Contact person: Prof.dr. M.C. Vos, Professor of Medical Microbiology, Infection Prevention, Dept of Medical Microbiology and Infectious Diseases*

*E-mail:* [*info.microbiologie.infectieziekten@erasmusmc.nl*](mailto:info.microbiologie.infectieziekten@erasmusmc.nl)

*We are grateful for your valuable comments which helped us to improve our manuscript. We sincerely appreciate the time you spent reviewing this manuscript. We revised our manuscript in accordance with your comments. Please find our responses to the comments below.*

**Reviewer #1:**

Introduction

1. Lines 48-49: Many research have been conducted until now in order to identify risk factors for SSI. However, one important point often overlooked is the importance of clinical setting. Indeed, risk factors for SSI could importantly differ between low income countries and high income countries.

A recent interesting work addressed this topic (Di Gennaro F, et al. Maternal caesarean section infection (MACSI) in Sierra Leone: a case-control study. Epidemiol Infect . 2020 Feb 27;148:e40. doi: 10.1017/S0950268820000370.) and could be cited, along with a brief paragraph discussing this point.

*We welcome this suggestion and consider the introduction as more rounded because of this addition. The following text was added to the introduction, and the suggested research article cited:*

*L51 – L53: “Risk factors in low-income countries also include unemployment and level of education due to the disparity in socioeconomic status [20].”*

Discussion

1. The antibiotic use as a risk factor for SSI is interesting but controversial. In fact, although many guidelines suggest to limit the use of the antibiotic prophylaxis at the day of the surgery, often antibiotics are prescribed several days before and after the surgical day.

In my opinion Authors should give a possible explanation for this very important result possibly based on their data.

*Although the data regarding the timing and administration of surgical antibiotic prophylaxis were not available for this study, the reviewer’s comment led us to further investigate the relationship between the occurrence of SSI and the time between J01 antibiotics prescription time and surgery. The occurrence of SSI seems to vary for different times between antibiotics prescription and surgery (Fig 1), but hypotheses regarding this relationship should be evaluated using case-control studies with specific data regarding the reason for the prescription, the type of antimicrobials and the timing of the administration thereof.*

**Fig 1. The occurrence of SSI for the time between the J01 antibiotics prescription start time and the start time of the surgery by decile.**

m, minutes; h, hours; d, days; #SSI, number of SSI occurrences; %SSI, percentage of SSI occurrences.

^1^The vertical axis starts at 75% to increase visibility.

^2^The horizontal axis stipulates the endpoints of the respective deciles of the distribution of the time between prescription and surgery.

*We added the following text to the discussion section to further share our insight with the readers:*

*L280 – L283: “Systemic antibiotics are typically prescribed to stabilise patients before undergoing surgery. A possible explanation for the increased occurrence of SSI associated with antimicrobials prescribed before surgery could be that these patients were not completely stabilised before surgery which increased their risk of SSI.”*

2. Did any data regarding the reason for surgical procedures were available? It is widely accepted that the cause of surgical procedure (infection, cancer, prosthetic implantation, etc) and the general clinical condition of patient (immunocompromised, severely undernourished, etc) are significant predictors of SSI. If these information are not available, this point should be included among limitations.

*We were hoping to include this information in the study but soon realised that these data were only available in free text field information, and there was no way to extract these data efficiently.*

*We added the following sentence to the Limitations section:*

*L288 – L290: “Surgeries were aggregated into three broad groups of surgical procedures which serve as a proxy for the reason for surgery but leads to the loss of information regarding the exact reasons for the surgery.”*

*L294-295: “A comprehensive list of variables related to the nutritional and immunological alterations of the patients was not included in the analyses as they were not available from the data.”*

**Reviewer #2:**

Methods

1. line 93: I think that could be useful to clarify how the nosocomial infection index was obtained, in particular which parameters where included to determine it.

*Thank you for bringing this to our attention. The references to the two articles which describe the nosocomial infection index, used as outcome variables in our study, are now provided immediately after the algorithm is mentioned for the first time. We also now refer to Streefkerk et al. in the text to make this referral clearer.*

2. Line 192-205: I think that this paragraph should be removed. It appears too technical and probably not necessary for the purpose of the article.

*This paragraph referred to was added as supplementary material (S2 Formulae) instead.*

Discussion

1. Lines 240-241: when you talk about antibiotic usage before surgery do you refer to their use in a broad sense or there are particular class of antibiotics related to a major risk? It could be interesting to examine which classes are related to a major risk for SSI.

*Antibiotics in J01 Anatomical Therapeutic Chemical were used this study. This definition is specified in the variable definition table, but the legend was not complete. We agree with the reviewer that delving deeper into the subclasses of antibiotics is of great importance, although not in the scope of this study.*

*The following changes were made to the manuscript:*

*L101-103: The following text was added to the methods section where the data are described: “Data regarding the prescription of antimicrobials, in the J01 class of the Anatomical Therapeutic Chemical (ATC) classification system [28], were also included.”*

*L161-L162: The following text was to the legend of the variable definition table: “ATC, Anatomical Therapeutic Chemical; WHO, World Health Organization.”*

2. Lines 256-257: there is a repetition, you just defined the general risk factors in lines 225-228

*Thank you, we have removed the repetition.*
